# Virtual reality technology for teaching neurosurgery of skull base tumor

**DOI:** 10.1186/s12909-019-1911-5

**Published:** 2020-01-03

**Authors:** Xuefei Shao, Quan Yuan, Daqing Qian, Zheng Ye, Gao Chen, Kang le Zhuang, Xiaochun Jiang, Yuelong Jin, Di Qiang

**Affiliations:** 1 Department of Neurosurgery, Yi-Ji Shan Hospital, Wannan Medical College, Wuhu, China; 2 Department of Imaging, Yi-Ji Shan Hospital, Wannan Medical College, Wuhu, China; 3 Clinical Skills Training Center, Wannan Medical College, Wuhu, China; 4 He Wang Lan digital ST CO.LTD, Hefei, China; 5 School of Public Health, Wannan Medical College, Wuhu, China; 6 Department of Dermatology and STD, Yi-Ji Shan Hospital, Wannan Medical College, No.2 West Road Zheshan, Jinghu district, Wuhu city, 241000 Anhui province China

**Keywords:** Virtual reality technology, Neurosurgery, Education

## Abstract

**Background:**

Neurosurgery represents one of the most challenging and delicate of any surgical procedure. Skull base tumors in particular oftentimes present as a very technically difficult procedures in the setting of neurosurgical teaching. Virtual reality technology is one of the most promising surgical planning tools. It can perform fast three-dimensional (3D) reconstruction of computed tomography (CT), magnetic resonance imaging (MRI) and other imaging data sets under conditions of virtual reality (VR). Surgical simulation can more intuitively understand the anatomical relationship of the surgical area in significantly greater detail.

**Methods:**

Thirty clinical undergraduates from the class of 2016 were randomly divided into two groups: the traditional teaching group and the virtual reality teaching group. After the study concluded, the teaching effectiveness was evaluated by combining basic theoretical knowledge, case analysis and questionnaire survey methods.

**Results:**

Comparative analysis between both groups showed the response effect of the virtual reality teaching group was better than that of the traditional teaching group (*P* < 0.05). There was also no difference between both groups in terms of the design of the surgical approach and the listing of surgical matters that required attention (*P* > 0.05).The results of theoretical knowledge assessment between both groups showed that the scores of basic theory, location, adjacent structure, clinical manifestation, diagnosis and analysis, surgical methods and total scores in the VR group exceeded those in the traditional teaching group (*P* < 0.05).

**Conclusions:**

This study showed that VR technology might improve neurosurgical skull base teaching quality, which should be promoted in the teaching of clinical subjects.

## Background

Neurosurgery represents one of the most challenging and delicate of any surgical procedure. Skull base tumors in particular oftentimes present as a very technically difficult procedures in the setting of neurosurgical teaching because of the small space available for surgical manipulations, the presence of adjacent vascular nerves, and the complicated surgical approach. Skull base neurosurgery not only depends on the microsurgical skills of the surgeon, but also familiarity with the knowledge of normal skull base anatomy and the inter-dependent relationships of the adjacent tumor site [1]. The teaching of neurosurgery has less class time, more technical content, and complicated anatomical structure to be taken into account, in addition to the related clinical manifestations, especially in the context of skull base tumors, which are difficult for students to understand and master [2].

Indeed, the development of neurosurgery depends on advances in the available surgical technology. The emergence of computed tomography (CT) and magnetic resonance imaging (MRI) has opened up a new era of neurosurgery. The clinical application of surgical microscopy, ventriculoscopy, intraoperative navigation, and multi-guided neurological monitoring has made the concept of minimally invasive neurosurgery widely applicable and a conceptual reality. Every leap forward in the technical levels of neurosurgery is inseparable from advances made in the available technology and the contributions made by novel instrumentation and associated supporting equipment. However, currently used approaches provides only a two-dimensional structural inspection, which is also a limitation. Moreover, it is difficult for the surgical novice to form three-dimensional information [3, 4]. Virtual reality technology is one of the most promising surgical planning tools. It can perform fast three-dimensional (3D) reconstruction of CT, and MRI and other imaging data sets under conditions of virtual reality, and can rotate, scale, segment, fuse and perform other reconstructed imaging tasks. This approach can also simulate various stereoscopic 3D images by using simulated surgical tools. Surgical simulation can more intuitively understand the anatomical relationship of the surgical area in significantly greater detail.In this current study, comparative assessment is made of the traditional forms of education with virtual reality techniques in the context of educational performance or capacity. This study evaluates the teaching results in the form of objective questions and subjective examination in virtual reality teaching and in the context of traditional mode teaching.

## Methods

### Student information

Thirty clinical undergraduates from the class of 2016 were randomly divided into two groups: 1) the traditional teaching group, and 2) the virtual reality teaching group. There were 30 students in the traditional teaching group, which was comprised of 12 males and 18 females, with a mean age of 20.63 ± 1.19 years. There were 30 students in the virtual reality teaching group, which was comprised of 16 males and 14 females, with a mean age of 20.47 ± 0.86 years. There was also no significant difference in terms of gender or age when comparing both groups (*P* > 0.05).

### Clinical case information

First, 10 cases of skull base tumors were admitted to the neurosurgery department of Yijishan Hospital between August 1, 2016 and August 1, 2018. Case data are described in Table 1.

**Table 1:** Clinical data about 10 patients

n	Sex	Age	Location	Pathology
1	F	44	Anteriorskull base	Meningioma
2	F	31	Middle skull base	Meningioma
3	M	52	Cerebellopontine angle	Schwannoma
4	F	61	Middle skull base	Meningioma
5	M	55	Middle skull base	Meningioma
6	M	48	Middle skull base	Meningioma
7	M	58	Cerebellopontine angle	Schwannoma
8	F	60	Cerebellopontine angle	Meningioma
9	F	51	Anterior skull base	Meningioma
10	M	56	Middle skull base	Meningioma

### Data collection

Data were collected from 10 patients that underwent CT scanning (SOMATOM Definition Flash, Siemens) and CT angiography. Methods applied in these scanning approaches included the following: KV: 120 KV; mAs: CARE Dose4D; and slice: 0.75 mm. Algorithms included the following: SAFIRE Kernel: H10f very smooth; Window: CT; Angio w: 700, L: 80, and FOV: 210×210 mm. Contrast agents were injected with use of iodophor at a dosing regimen of 350 mg/ml and 5 ml/s. Magnetic resonance examination (MRI; MAGNETIOM Avanto Dot, Siemens) sequence 1: t1_mpr_sag_p2 + c; matrix: 256×228; Flip: 15^0^; TR: 1910 MS; TE: 3.07 ms; T1: 1100 ms; and ThK: 1 mm. BW: 130 Hz/Px; and FOV: 234×250 mm. Sequence 2: TOF-3D: Matrix: 256×196; Flip: 25^0^; TR: 25 ms; TE: 7 ms; ThK: 0.8 mm; BW: 121 Hz/Px; and FOV: 183 × 230 mm. After scanning, the aggregated data was imported using the 3D-Slicer software program. Threshold, Islands, Draw Tube, Logical operators, surface cut and other functional modules in the Segment Editor function were used for threshold segmentation. Finally, the generated modeling data were exported to STL formatted files, and the STL files were imported into Microsoft Hololens® glasses using the Unity-3D development software program.

### Teaching methods

In the same teaching time, the traditional teaching group combined literature-based learning (LBL) with teaching mode of the text, images, animation and video in the form of multimedia data sets, and problem-based teaching (PBL) and case-based teaching (CBL). Virtual reality teaching used real case images as used by Microsoft Hololens® glasses.

### Teaching effectiveness evaluation

After the study had concluded, the teaching effectiveness was evaluated by combining basic theoretical knowledge, case analysis and questionnaire survey methods. Among them, the basic theoretical knowledge test included the basic theory of skull base structure, neurological specialty examination and clinical manifestations. The case analysis test included diagnosis and analysis, neuroimaging data analysis, and treatment plan formulation. The questionnaire survey included the learning interest, demonstration of knowledge and comprehension, clinical thinking ability, and actual clinical work efforts. Whether a given ability was improved upon, and whether there was evidence of achieving satisfaction in the teaching method was also determined.

### Statistical analysis

The SPSS18.0 statistical software program was used for data analysis. Metrological data were expressed by the mean ± one standard deviation about the mean. The Student’s t-test was used for inter-group comparisons. Counting data were expressed by examples or percentages, and the Chi-squared (× ^2^) test was used for inter-group comparisons. Inspection level.

## Results

### Information collection of one case by virtual reconstruction

**Fig. 1:**
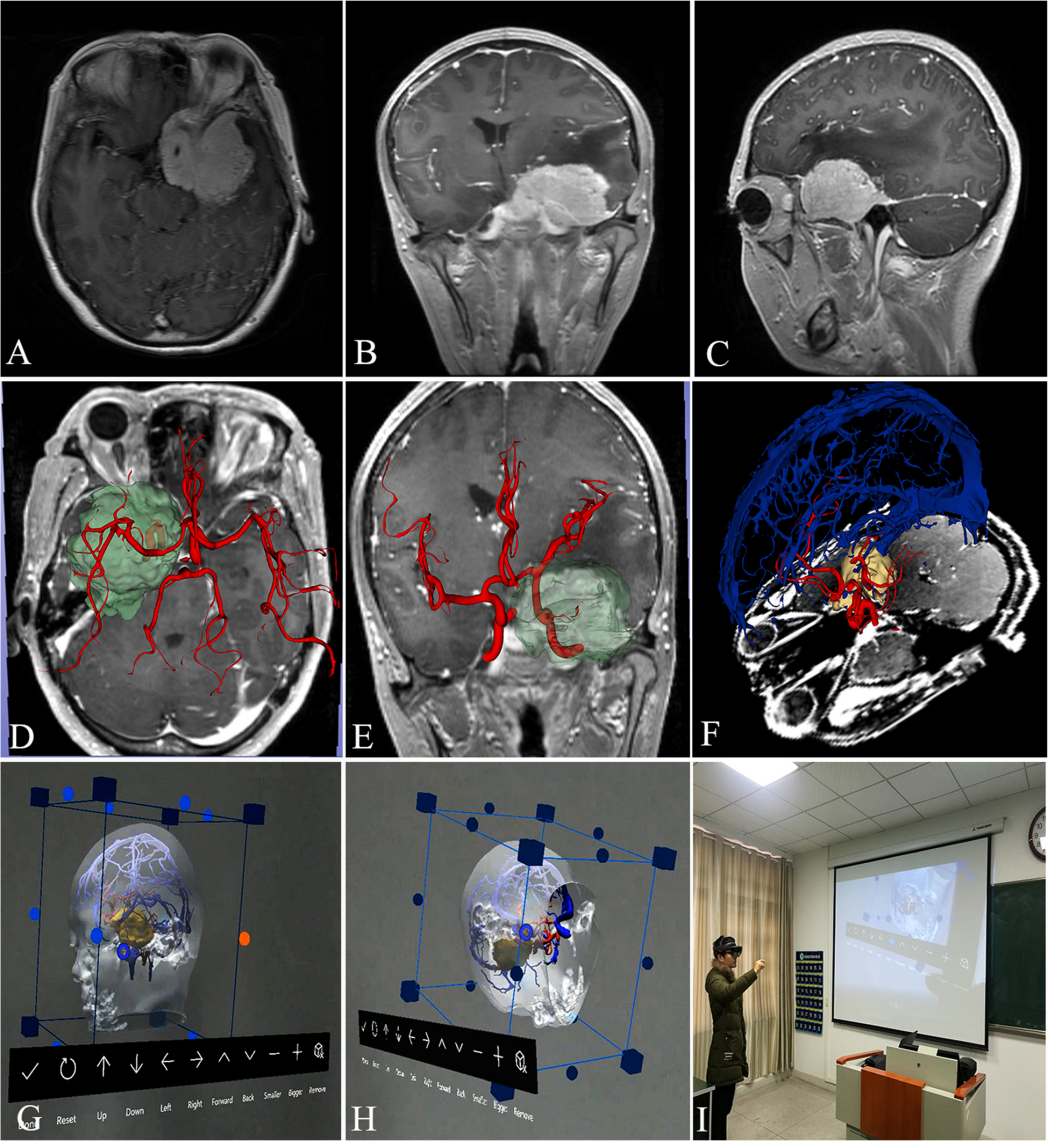
Virtual reconstruction of a left sphenoid ridge meningioma in one patient. **a-c** MRI results. **d**-**f** 3D display of MRI. **g**, **h** The images viewed by the Microsoft Hololens glasses. **h** The image of one student using the Microsoft Hololens glasses


The MRI result of a 44-year-old female patient with a left sphenoid ridge meningioma was used to perform 3D reconstruction. As shown in Fig. 1, enhanced MRI revealed that the tumor was located internal and external to the sphenoid ridge and enclosed the internal carotid artery (Fig. 1a-c). After scanning, the aggregated data was imported into the 3D-Slicer software program. Then, tumor, peritumoral structures, arterial vessels and venous vessels were three-dimensionally showed in the MRI (Fig. 1d-f). The STL files were into Microsoft Hololens glasses using the Unity-3D development software program. These images were viewed using the Microsoft Hololens glasses (Fig. 1g and h), and one student was using the Microsoft Hololens glasses (Fig. 1i). The whole process of virtual reconstruction was showed in Additional file 1: Video S1.

**Additional file 1: Video S1.** Video of virtual reality technology in Teaching.


### The results of subjective questionnaire survey

The contents of subjective questionnaire survey were designed and showed in Table 2. All students in this study finished the subjective questionnaire survey, and the comparative analysis between both groups showed whether or not the VR group could identify the surrounding tumors, intuitively understand the skull base structure, combine the location of the tumors with associated clinical manifestations, and improve the conversion from 2D to 3D thinking. This analysis also determined whether the teaching method could improve learning interest and whether the teaching method was satisfactory. As shown in Table 3, the response effect of VR group was better than that of the traditional teaching group (*P* < 0.05). There was also no difference between both groups in terms of the design of the surgical approach and the listing of surgical matters that required attention (*P* > 0.05).

**Table 2:** Subjective Questionnaire Results

Question	useless at all	useless	no idea	useful	very useful
1. To be intuitively understand the skull base structure?	①	②	③	④	⑤
2. To be identify the structure around the tumor?	①	②	③	④	⑤
3. To be combine the location of tumors with clinical manifestations?	①	②	③	④	⑤
4. To be improve the transformation from 2D to 3D?	①	②	③	④	⑤
5. To be designed surgical approach	①	②	③	④	⑤
6. To be list the attentions in operation?	①	②	③	④	⑤
7. To be improve learning interest by this teaching?	①	②	③	④	⑤
8. To be satisfied with the teaching method?	①	②	③	④	⑤

Key:agree ①useless at all, ②useless, ③no idea, disagree ④useful, ⑤very useful

**Table 3:** Comparison of two groups of students learning interest and research thinking ability

Item	Traditional teaching		VR teaching		*x*^2^	*P*
	agree	disagree	agree	disagree		
1	13 (43.3)	17 (56.7)	22 (73.3)	8 (26.7)	5.554	0.018
2	13 (43.3)	17 (56.7)	28 (93.3)	2 (6.7)	17.330	0.000
3	15 (50.0)	15 (50.0)	23 (76.7)	7 (23.3)	4.593	0.032
4	11 (36.7)	19 (63.3)	25 (83.3)	5 (16.7)	13.611	0.000
5	10 (33.3)	20 (66.7)	16 (53.3)	14 (46.7)	2.443	0.118
6	16 (53.3)	14 (46.7)	22 (73.3)	8 (26.7)	2.584	0.108
7	14 (46.7)	16 (53.3)	26 (86.7)	4 (13.3)	10.800	0.001
8	12 (40.0)	18 (60.0)	24 (80.0)	6 (20.0)	10.000	0.002

### Comparison of the results of theoretical knowledge assessment between both groups of students

The results of theoretical knowledge assessment between both groups showed that the scores of basic theory, location, adjacent structure, clinical manifestation, diagnosis and analysis, surgical methods and total scores in the VR group exceeded those in the traditional teaching group (*P* < 0.05; Table 4).

**Table 4:** Comparison of the results of theoretical knowledge assessment between two groups of students

Group	Basic theory (50)	Tumor location (10)	Adjacent structure of tumor (10)	Clinical manifestation (10)	Diagnosis and analysis (10)	Operative approach (10)	Total score (100)
Traditional teaching	35.77 ± 2.97	5.43 ± 1.10	5.70 ± 1.26	5.6 ± 1.19	5.33 ± 1.25	5.77 ± 0.97	63.60 ± 3.81
VR teaching	41.23 ± 3.06	7.20 ± 1.16	7.03 ± 1.25	7.27 ± 1.17	6.97 ± 1.27	7.37 ± 1.13	77.07 ± 4.00
*t*	7.026	6.051	4.117	5.460	5.033	5.881	13.352
*P*	0.000	0.000	0.000	0.000	0.000	0.000	0.000

## Discussion

Traditional teaching methods in university medical colleges are conventionally based on LBL. This approach depends on oral instruction and blackboard demonstrations that are combined with the assistance of teaching tools that might include a model, specimens, autopsy demonstration, and animal experimentation. An autopsy is often undertaken to deepen a student’s understanding and improve the student’s practical ability in the “real world”.

In recent years, multimedia teaching has integrated information such as text, imaging, sounds, animation and video into a holistic whole, which combines PBL, CBL and other novel teaching methods, including lively and vivid forms of expression that have emerged as the mainstay of modern practical teaching methods.

Autopsy is a traditional method of surgical education. Neurosurgery seeks to understand cranio-cerebral anatomy through autopsy, which is both real and spatial. However, the number of specimens is not only scarce, but also needs to be equipped with perfect neurosurgical anatomical experimental instruments that can only be achieved across just a few medical colleges. Moreover, autopsy cannot be repeated, and it is difficult to homogenize this approach with clinical cases [5, 6]. Recent popular 3D printing technology can replicate individual and realistic models; however, it is difficult to simulate different organizational structures without suitable printing materials, and the cost of this approach is too high for most teaching hospitals or medical colleges [7].

Traditional CT, MRI, MRA and MRV approaches are routine examinations that are undertaken prior to skull base surgery. CT is helpful in displaying skull base bone structure and plain scan MRI can show brain tissue, the ventricle and other structures; Moreover, following enhancement plain scan MRI can clearly show tumors and some nerve tissues and blood vessels. In addition, MRA can show the arteries and other blood vessels, and MRV can show the veins and other blood vessels. All types of imaging technology have their own unique characteristics, advantages and application scope. Imaging also represent the main approach at teaching skull base neurosurgery [8]. However, it is difficult for students to understand complex 3-D craniocerebral anatomy by simple two-dimensional Atlas [9].

Eftekhar achieved good results in patients with preoperative anterior aneurysms using VR technology [10]. Bernard F developed of 3-dimensional virtual reality video tutorials in the French neurosurgical residency program [11]. Virtual reality uses multi-image fusion technology to synthesize imaging information and provide intuitive and realistic images that can complement the advantages of each approach and optimally maximize the display of medical imaging data. Virtual reconstruction of digital information restores real cases, and can cut and observe from different angles and planes. Anatomical morphology can be observed by sequentially stripping, and the adjacent relationship of major organs can be observed across different planes.

In this study, 10 cases of skull base tumors were reconstructed by simulation that was combined with realistic spectacles that enabled students to visually and accurately judge tumor characteristics, skull base and adjacent anatomical structures, analyze difficulties encountered during surgery and the corresponding measures, and thus formulate individual optimized surgical methods and approaches. Its advantages include: (1) defining the relationship between tumors and blood vessels. For tumors of the anterior or middle skull base, including sphenoid ridge meningiomas, it is understood that the middle cerebral artery is located in the tumors or on the tumor surface (Fig. 1f and g). (2) Determination of the relationship between tumors and nerves. (3) Selection of the operative methods and approaches. By observing the spatial position and shape of the tumors, measuring the distance and angle between tumors and the cortex and skull base, and simulating the intracranial structures that are observed in different surgical approaches, it is helpful to compare the advantages and disadvantages of different surgical approaches and pathways, and to determine optimal individualized surgical approaches and pathways. Especially for giant tumors straddling the anterior, middle and posterior skull base or deep tumors like petroclival tumors, the approach selected is particularly important. It is the greatest advantage of this teaching method that the operator can repeatedly simulate, compare and contrast in the VR environment pre-surgery and then select the best scheme. This information is conducive to identifying and protecting these important vessels during surgery, and to reduce the occurrence of intraoperative complications.

Virtual reality technology not only arouses the interest of students to learn, it also consolidates and enables visualization of the abstract content, which provides the student a deeper memory of the learning experience, providing useful convenience for the instructor and greatly improving teaching quality. It can also simultaneously alleviate the lack of cadaver specimens, thus reducing teaching costs and compensating for a shortfall in teaching conditions. De Faria used the virtual imaging technique of brain anatomy to teach, and achieved good results [12].

Since VR technology has the characteristics of immersion, interaction and imagination, its application in the experimental teaching of medical education has unique advantages in training medical vocational skills.

Using VR technology, students can use it for detailed observations to obtain first-hand concepts and knowledge, which can greatly improve understanding and mastery by the student. Virtual technology can also overcome the limitation of time, combining classical clinical cases with preoperative, intraoperative and postoperative results, through VR technology, which can be presented to students in a very short period of time for observation. The application of VR technology strengthens the critical thinking of students, and provides innovative learning activities. Virtual reality technology realizes that students and the virtual environment interact and influence each other in two important aspects. First, it creates an environment of “self-learning,” which is replaced by the traditional learning mode of “teaching to promote learning” by students themselves. Second, interaction with information and the environment is a novel approach to acquire new knowledge and skills. Students are active observers in the VR environment. They need to constantly explore, analyze, judge and summarize their thinking activities in this learning environment. Moreover, the excellent interaction provided by virtual technology enables computer teaching systems to provide students with the necessary feedback, so that students can become independent and personalized [13].

This study showed that VR technology might improve neurosurgical skull base teaching quality, which should be promoted in the teaching of clinical subjects.VR technology is the product of the intersection, infiltration and integration of multi-disciplinary fields. There is still a long way to go to make it a widely accepted by medical education, including the development and innovation of software and hardware, and the strengthening of the great relationship with clinical practice.

## Conclusions

This study showed that VR technology might improve neurosurgical skull base teaching quality, which should be promoted in the teaching of clinical subjects. Virtual reality can be enlarged, rotated and shifted arbitrarily, and can select a three-dimensional model of any organizational structure in the human body and use information from the digital virtual human. The limitation of the study lies in whether students will communicate privately after class, which will lead to the decline of learning interest of traditional teaching groups, and whether it will have a minor impact on the conclusions of the study. In the future research work, we not only to increase the sample size, but also to carry out two different teaching methods in different schools or campuses in order to have more authentic research results.

## Data Availability

The datasets used and/or analyzed during the current study are available from the corresponding author on reasonable request.

## Abbreviations

3D: Three-dimensional
CBL: Case-based teaching
CT: Computed Tomography
LBL: Literature-based learning
MRA: Magnetic resonance artery
MRI: Magnetic resonance imaging
MRV: Magnetic resonance vein
PBL: Problem-based teaching
VR: Virtual reality

## References

[1] Gagliardi F, Chau AM, Mortini P, Caputy AJ, Gragnaniello C (2018). Skull Base Neuroendoscopic training model using a fibrous injectable tumor polymer and the Nico myriad. J Craniofac Surg.

[2] Hanrahan J, Sideris M, Tsitsopoulos PP, Bimpis A, Pasha T, Whitfield PC, Papalois AE (2018). Increasing motivation and engagement in neurosurgery for medical students through practical simulation-based learning. Ann Med Surg (Lond).

[3] Kim DH, Kim Y, Park JS, Kim SW (2019). Virtual reality simulators for endoscopic sinus and Skull Base surgery: the present and future. Clin Exp Otorhinolaryngol.

[4] Hendricks BK, Patel AJ, Hartman J, Seifert MF, Gadol AC (2018). Operative anatomy of the human skull: a virtual reality expedition. Oper Neurosurg (Hagerstown).

[5] Benet A, Rincon-Torroella J, Lawton MT (2014). Novel embalming solution for neurosurgical simulation in cadavers. J Neurosurg.

[6] Romero AD, Zicarelli CA, Pinto FC, Pasqualucci CA, Aguiar PH (2009). Simulation of endoscopic third ventriculostomy in fresh cadaveric specimens. Minim Invasive Neurosurg.

[7] Weinstock P, Prabhu SP, Flynn K (2015). Optimizing cerebrovascular surgical and endovascular procedures in children via personalized 3D printing. J Neurosurg Pediatr.

[8] Mason WT, Strike PW (2003). See one, do one, teach one--is this still how it works? A comparison of the medical and nursing professions in the teaching of practical procedures. Med Teach.

[9] Johnson AM (1990). The speed of mental rotation as a function of problem-solving strategies. Percept Mot Skills.

[10] Eftekhar B (2017). Smartphone as a remote touchpad to facilitate visualization of 3D cerebral angiograms during aneurysm surgery. J Neurol Surg A Cent Eur Neurosurg.

[11] Bernard F, Gallet C, Fournier HD, Laccoureye L, Roche PH, Troude L (2019). Toward the development of 3-dimensional virtual reality video tutorials in the French neurosurgical residency program. Example of the combined petrosal approach in the French College of Neurosurgery. Neurochirurgie.

[12] de Faria JW, Teixeira MJ (2016). de Moura Sousa Júnior L, Otoch JP, Figueiredo EG. Virtual and stereoscopic anatomy: when virtual reality meets medical education. J Neurosurg.

[13] Stepan K, Zeiger J, Hanchuk S, Del Signore A, Shrivastava R, Govindaraj S, Iloreta A (2017). Immersive virtual reality as a teaching tool for neuroanatomy. Int Forum Allergy Rhinol.

